# Intraoperative Radiotherapy With INTRABEAM: Technical and Dosimetric Considerations

**DOI:** 10.3389/fonc.2018.00074

**Published:** 2018-03-26

**Authors:** Anil Sethi, Bahman Emami, William Small, Tarita O. Thomas

**Affiliations:** Loyola University Medical Center, Maywood, IL, United States

**Keywords:** intrabeam, spherical, flat, surface applicators, dosimetry

## Abstract

**Purpose:**

We evaluate dose characteristics and clinical applications of treatment accessories used in intraoperative radiotherapy (IORT) and make site-specific recommendations for their optimal use.

**Methods and materials:**

Dose measurements were performed for a low energy (50 kV) X-ray INTRABEAM source. For spherical, flat, surface, and needle applicators, the following dosimetric parameters were measured: depth-dose (DD) profiles, surface dose (Ds), output factors (OF), and target dose homogeneity (DH). Optical density versus exposure calibration films were employed to obtain 2-dimensional dose distributions in planes parallel and perpendicular to beam direction. Film results were verified *via* repeat dose measurements with a parallel-plate ionization chamber in a custom water tank. The impact of applicator design on dose distributions was evaluated.

**Results:**

Spherical applicators are commonly used for treating the inner-surface of breast lumpectomy cavity. Flat and surface applicators provide uniform planar dose for head and neck, abdomen, and pelvis targets. Needle applicators are designed for kypho-IORT of spinal metastasis. Typically, larger applicators produce a more homogeneous target dose region with lower surface dose, but require longer treatment times. For 4-cm diameter spherical, flat, and surface applicators, dose rates (DR) at their respective prescription points were found to be: 0.8, 0.3, and 2.2 Gy/min, respectively. The DR for a needle applicator was 7.04 Gy/min at 5 mm distance from the applicator surface. Overall, film results were in excellent agreement with ion-chamber data.

**Conclusion:**

IORT may be delivered with a variety of site-specific applicators. Appropriate applicator use is paramount for safe, effective, and efficient IORT delivery. Results of this study should help clinicians assure optimized target dose coverage and reduced normal tissue exposure.

## Introduction

Intraoperative radiotherapy (IORT) delivers a large tumoricidal dose to a well-defined target at the time of surgery while simultaneously minimizing exposure to nearby normal structures (1, 2). Compared to external beam radiotherapy, the advantages of IORT are: potential for dose escalation, reduced overall treatment time, and enhanced patient convenience. IORT may be delivered with either an external beam of low-energy electrons (3), kV X-rays (4, 5), or *via* a miniaturized X-ray tube used as radiation source in electronic brachytherapy (6).

**Figure 1:**
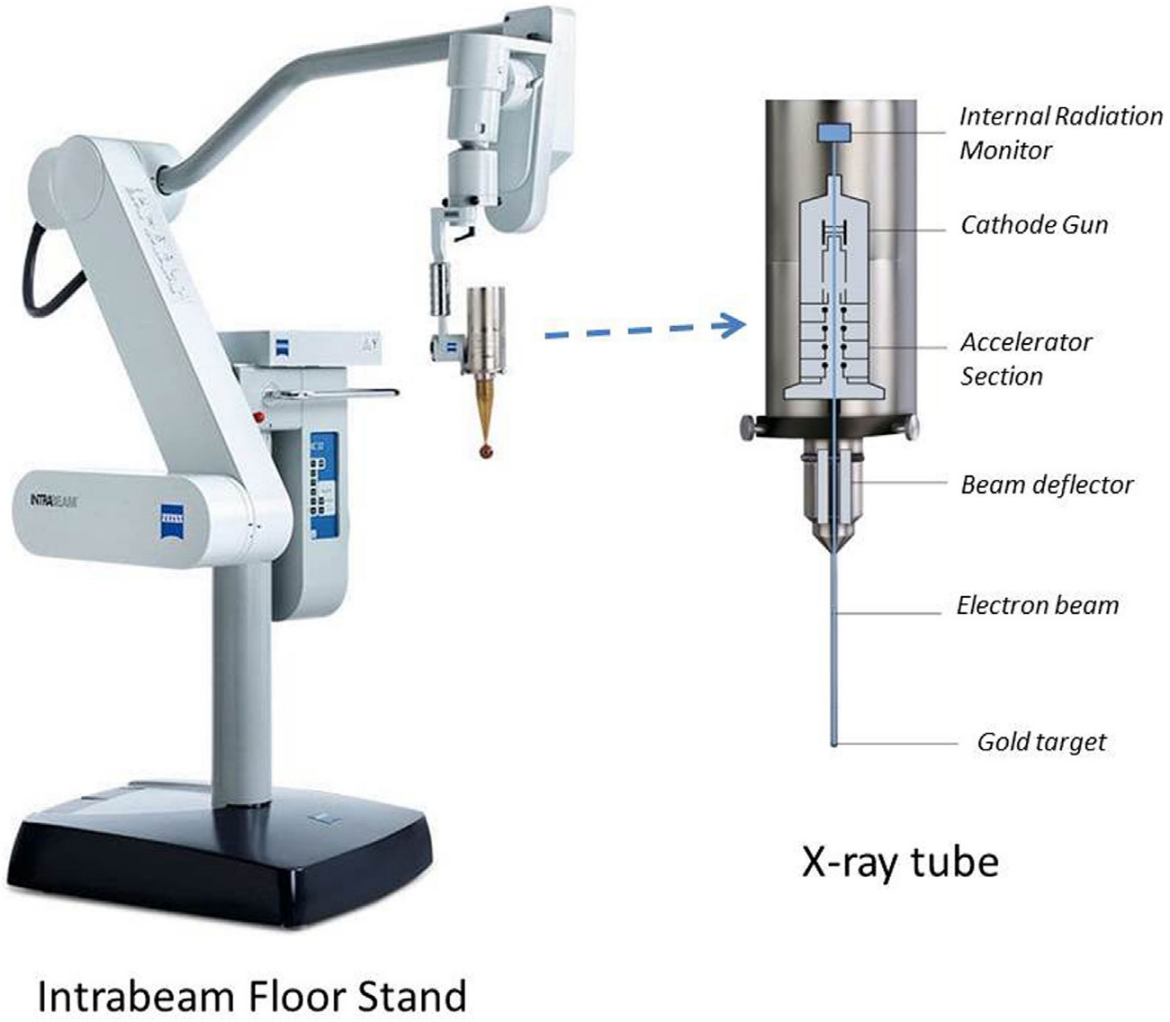
Intrabeam stand and X-ray source shown with spherical applicator attachment. The floor stand provides full flexibility of movement for the X-ray tube with millimeter precision in six dimensions. The floor stand weighs 275 kg and has dimensions of 74 cm × 194 cm × 150 cm (W × H × L) in transport position. Also shown are internal components of the X-ray source (dimensions: 7 cm × 11 cm × 17 cm; weight: 1.6 kg).

The IORT investigated in this report is based on an INTRABEAM X-ray source, PRS 500 (INTRABEAM, Carl Zeiss Surgical, Oberkochen, Germany) emitting low-energy (50 kV) photons at a high dose-rate (Figure 1) (4, 5, 7–12). The IORT’s appeal lies in its ability to deliver a large dose (10–20 Gy) to the target volume with rapid dose fall-off and hence limited exposure to adjacent organs at risk. Furthermore, with appropriate precautions, the low energy X-rays result in minimal radiation risk to the operating room personnel. Recent IORT advances, such as the availability of novel treatment applicators to shape radiation dose to a desired target volume have resulted in tremendous gains in its clinical applications (13). Currently, some of the more common treatment sites for IORT include: breast, head and neck, brain, abdomen, pelvis, rectum, sarcoma, and spine.

At Loyola University Medical Center, we have commissioned and clinically used the INTRABEAM system with spherical, flat, surface, and needle applicators. Owing to their symmetric shape, spherical applicators are used to deliver a uniform dose at the inner-surface of the breast lumpectomy cavity. The surface and flat applicators are used when a constant dose is desired at a given tissue-depth: 0 and 5 mm, respectively. The needle applicator is designed for kypho-IORT (spine metastasis). These applicators are available in a range of sizes (diameter: 0.5–6 cm).

Although spherical applicators have been in use for a long time for breast IORT, the flat, surface, and needle applicators have recently become available and their clinical applications are becoming more popular. However, at present, there is a lack of available data for these applicators, which may limit their clinical use. Having a thorough understanding of IORT dose distribution is essential for safe, effective, and efficient treatment delivery. In this paper, we report on the dosimetric characteristics of each applicator, including, dose rate (DR), depth-dose (DD), dose homogeneity (DH), and treatment time. In addition, practical guidelines are provided for the optimal use of each applicator for various treatment sites. However, it is our recommendation that each institution intending to practice IORT must validate the dosimetric data of their equipment prior to clinical use.

## Materials and Methods

All measurements were performed for an INTRABEAM 50-kV X-ray source fitted with either a spherical, flat, surface, or needle applicator to produce desired spherical or planar dose (Figure 1) (10). At our institution, the physician performing IORT may choose from any of the following treatment accessories: five spherical applicators (diameter: 3–5 cm in 0.5 cm increment), six flat applicators (diameter: 1–6 cm in 1 cm increment), and four surface applicators (diameter: 1–4 cm in 1 cm increment). For each applicator, DD profiles, surface dose, output factors (OFs), and DH were measured:

(a) DD profiles are measured as change in DR with depth in a phantom.
(b) Surface dose (Ds) is defined as the dose at the target surface in contact with applicator tip.
(c) Output factor (OF) refers to the delivered dose at the prescription point in 1 min (Gy/min). The prescription point depends on the applicator type selected: surface of spherical applicator, 5 mm depth in phantom for flat applicator, phantom surface for surface applicator, and 5 mm from the tip for needle applicator.
(d) DH is defined as the variation in dose (*D*_max_*/D*_min_) in the beam direction in the target region of interest.

**Figure 2:**
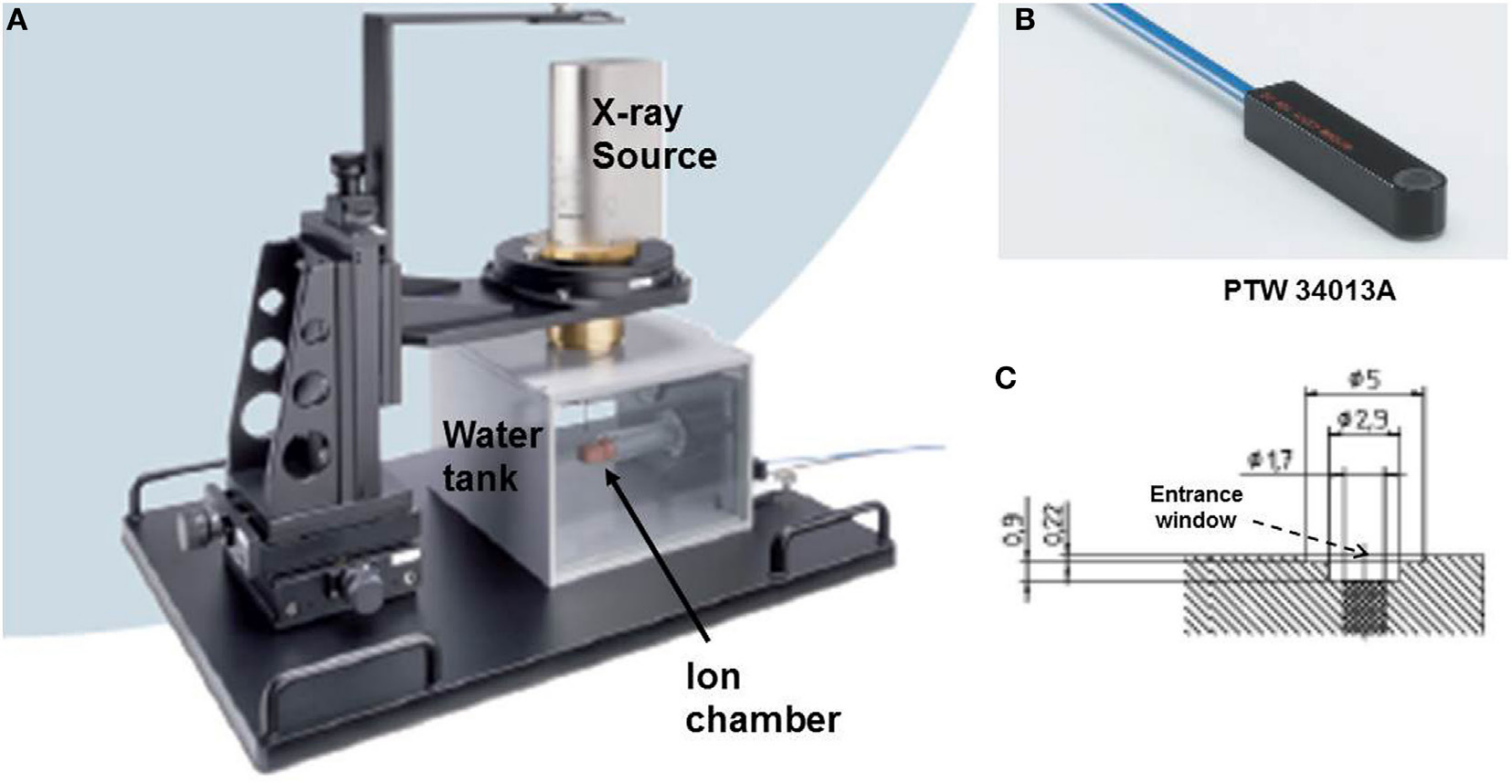
Measurement setup for dose output factor, OF and depth dose profiles, DD. **(A)** Water tank showing positions of ion chamber and X-ray source; **(B)** PTW34013A thin-window parallel plate ion chamber used for dose measurements; **(C)** a schematic diagram of the parallel plate ion chamber showing beam entrance window (thickness = 0.22 mm).

Output factors in terms of absolute DR (Gy/min) and DD profiles were measured in a water tank (Figure 2A) with a suitable thin-window parallel plate ion chamber (PTW 34013A, Physikalisch Technische Werkstaetten, Freiburg, Germany) (Figure 2B) (14). The ion chamber was connected to a PTW UNIDOS electrometer T10010 to record measured charge. The water-phantom available from Zeiss (Carl Zeiss Surgical, Oberkochen, Germany) allows precise positioning of applicator tip relative to ion chamber for accurate dose output and dose distribution measurements.

The measured DR (*Gy/min*) at a specific depth *z* in water can be written as:

(1) $$\text{DR}(z)=N_{\text{k}}Q(z)C_{\text{TP}}k_{\text{Q}}k_{\text{elec}}$$

where *N*_k_ is the ion chamber calibration factor (*Gy/nC*), *Q(z)* is the ionization charge (C) collected in 60 s for the chamber located at depth *z* in water, *C*_TP_ is the correction factor for room temperature (*T*) and pressure (*P*) at the time of dose measurement, *k*_Q_ is the beam quality correction factor, and *k*_elec_ is the electrometer calibration factor. The measured DR is plotted as a function of depth *z* to obtain depth dose profiles (DD).

DH data were obtained from the measured DD profiles. Surface-dose (Ds) data were acquired with a film dosimeter. Whenever possible, film data were confirmed with ion-chamber measurements.

**Figure 3:**
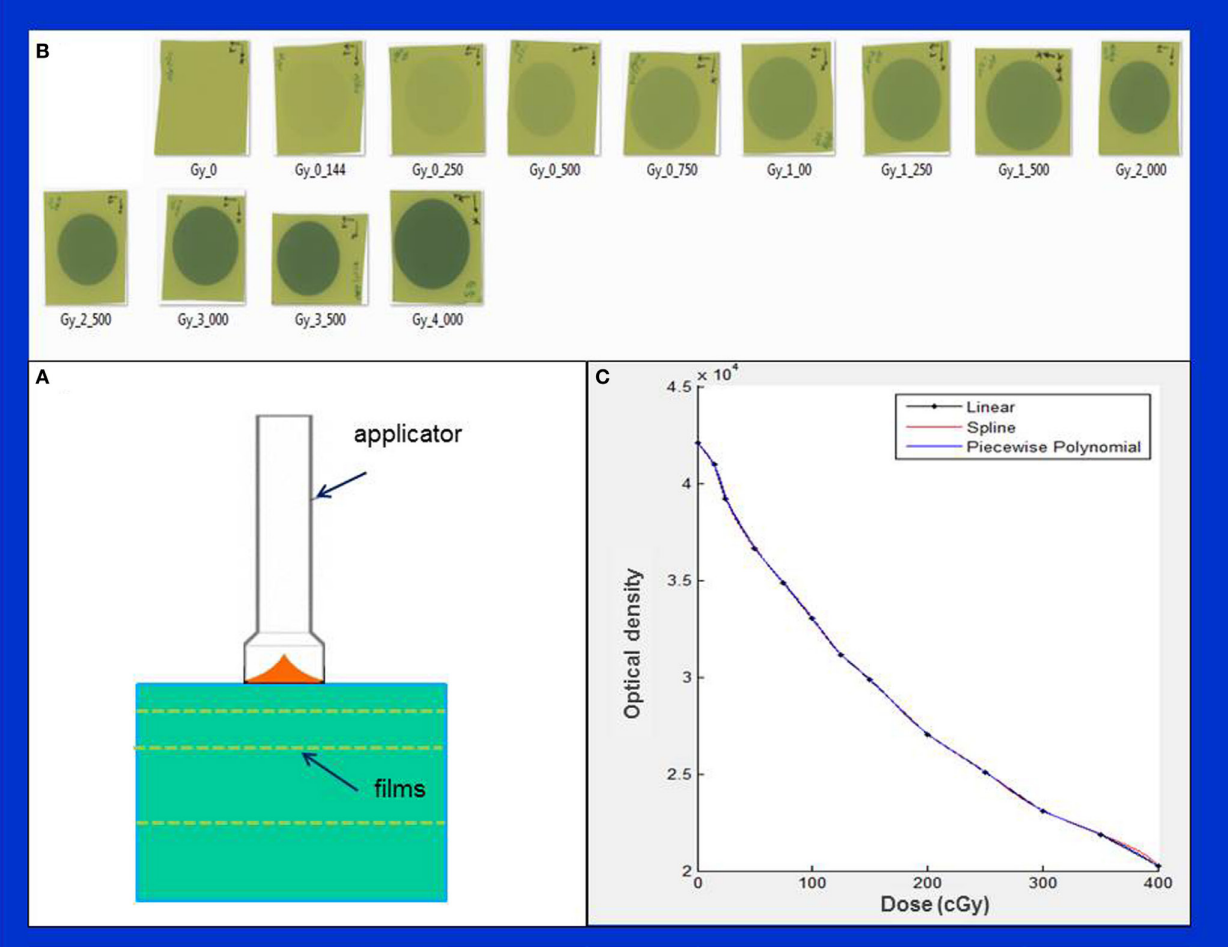
Determination of film sensitometric (H&D) curve. **(A)** Measurement setup showing X-ray source, phantom slabs, and EBT3 films; **(B)** a batch of EBT3 films irradiated in the dose range: 0–400 cGy; **(C)** H&D curve showing variation of film optical density with dose.

The spherical applicator’s isotropic dose distribution is easily mapped with an ion chamber. The dose characteristics and treatment times for a needle applicator are available from a look-up table available with Zeiss, Inc. (Carl Zeiss Surgical, Oberkochen, Germany). Dose distributions generated with flat and surface applicators, on the other hand, are more intricate, requiring the use of a 2-dimensional film dosimeter. Films are an efficient tool to measure planar dose distribution. We used Gafchromic EBT3 films (International Specialty Products - ISP, Wayne, NJ, USA) sandwiched between slabs of water equivalent phantom (Plastic water, CIRS, Norfolk, VA, USA) (15) (Figure 3A). The films were aligned in both parallel and perpendicular orientations relative to the radiation beam (only films in perpendicular orientation are shown in Figure 3A). For each measurement, the X-ray source was oriented vertically with the end of the applicator in contact with the phantom surface (Figure 3A).

First, we established a film characteristic or sensitometric curve (also known as Hurter and Driffield or H&D curve), which relates film optical density with film exposure or dose (Figures 3B,C). This was done by irradiating several films from the same batch to a known dose (range: 0–4 Gy) delivered at 5 mm depth from the phantom surface (Figure 3B). Absolute calibration of IORT source was validated *via* Eq. 1 given above. Since the EBT3 film response is known to be highly sensitive to environmental conditions, all necessary film precautions were observed, for example, handling of films with tweezers and latex gloves (15–19). Furthermore, following each irradiation, films were allowed to self-develop for at least 24 h to stabilize dose response. An EPSON 11000XL PRO flatbed scanner was used with films placed at its center in the portrait orientation. The red color channel was used and film pixel values were converted to dose using the sensitometric (H–D) curve. Film scanning and analysis software, RIT ver. 6.4 (Radiological Imaging Technology, Colorado Springs, CO, USA) was used to generate 2-dimensional dose distributions. With each applicator, films were irradiated to deliver 1 Gy dose at the prescription depth. All film results (DD profiles, OF, etc.) were verified with parallel-plate ion chamber measurements in the water phantom.

## Results

### Spherical Applicators

**Figure 4:**
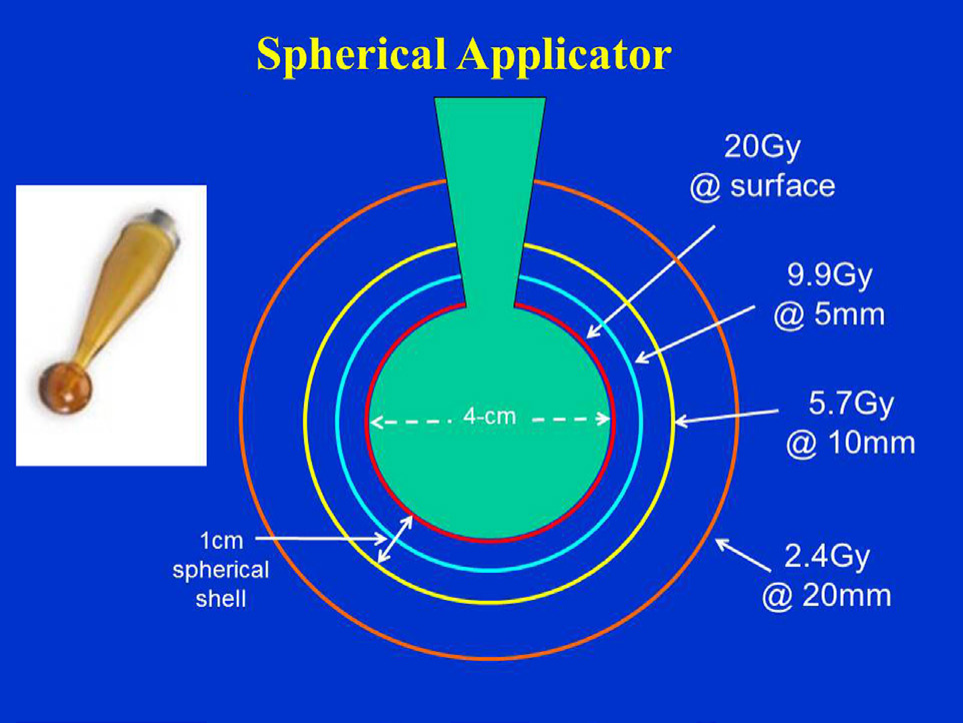
Dose distribution produced by a 4 cm diameter spherical applicator. Doses are shown at the applicator surface and at 5, 10, and 20 mm distance from it. Dose homogeneity was evaluated in a 1 cm thick spherical shell surrounding the applicator. Applicator shown in inset.

**Figure 5:**
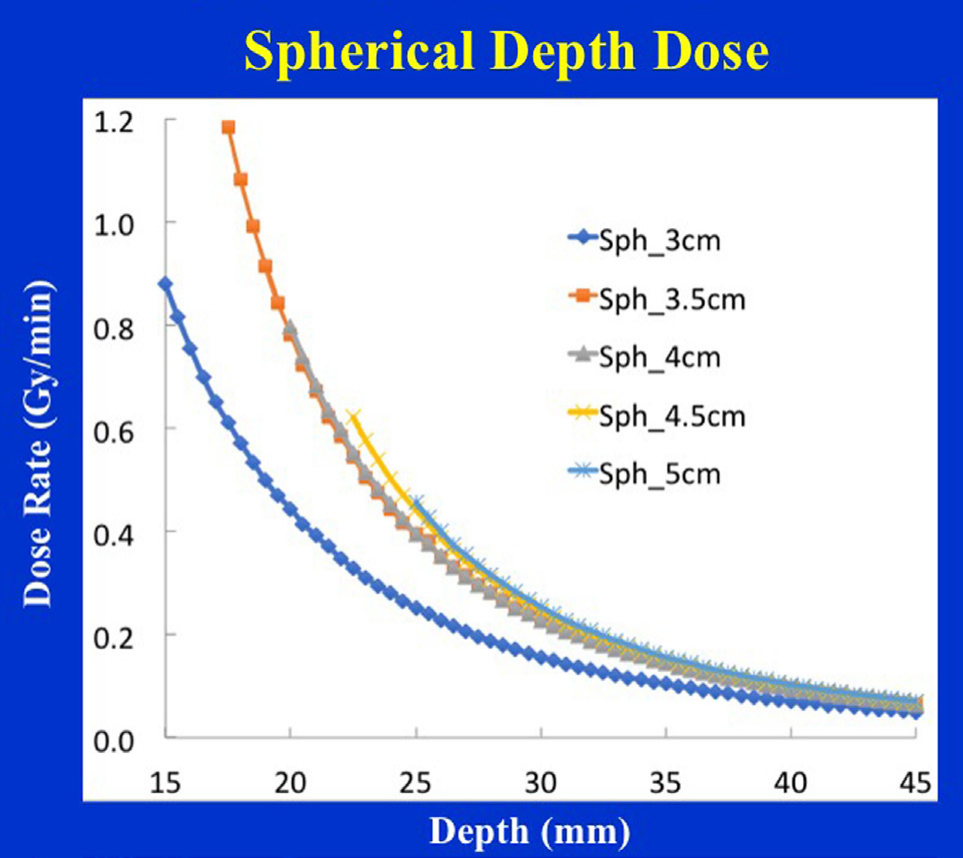
Spherical applicator depth-dose (DD) profiles for a range of diameters (3–5 cm) plotted as a function of the distance from the X-ray source. Note that each depth dose profile begins at the applicator surface, for example, DD profile for the 3 cm applicator begins at 1.5 cm from the X-ray source, etc.

Figure 4 shows typical dose distribution produced by a 4 cm diameter spherical applicator. A 20 Gy dose was prescribed at the applicator surface (or the lumpectomy cavity-inner surface). For effective skin sparing, the applicator surface must be at least 1 cm depth from the skin-surface. In the present case, the 20 Gy dose at the applicator surface would result in 5.7 Gy skin dose. Target DH, defined as the ratio of maximum and minimum doses (*D*_max_*/D*_min_), was evaluated in the radial direction within a 1 cm thick spherical shell surrounding the applicator (as indicated in the figure). For the 4 cm spherical applicator, the measured DR at the applicator surface was 0.8 Gy/min, which resulted in a treatment time of 25 min. At 1 cm from the applicator surface, the DR was 0.23 Gy/min, corresponding to a DH = 3.5. In general, larger applicators require longer treatment time due to lower surface DR, but yield superior target dose homogeneity (DH values closer to unity) or a slower dose fall-off in the target region. Due to its unique design features, the 3-cm diameter spherical applicator has a lower surface dose and slower dose fall-off compared to the 3.5 cm applicator. This results in somewhat longer than expected treatment times with the 3 cm applicator (Table 1). Figure 5 shows the surface DRs and dose fall-off with depth (DD profiles) for spherical applicators with diameters ranging from 3 to 5 cm (treatment times: 17–44 min). For each applicator, the DD curve starts at the applicator surface, for example, 15 mm from the X-ray source for a 3 cm applicator, 20 mm from the X-ray source for a 4 cm applicator, etc. Radial DH, in the 1 cm spherical shell surrounding each applicator (presumed target region) ranged between 3 and 4.

**Table 1:** Dosimetric characteristic of various intraoperative radiotherapy applicators used with INTRABEAM.

Dosimetric comparison of applicators				

Applicator type and diameter	Rx dose (Gy)	Surface dose, Ds (Gy)	Dose homogeneity (DH) (*D*_max_/*D*_min_)	Treat time (min)
Sphere 3 cm	20	20	3.5	23
Sphere 4 cm	20	20	3.5	25
Sphere 5 cm	20	20	2.9	44
Flat 2 cm	10	25.1	2.5	13
Flat 4 cm	10	19.4	1.9	32
Flat 6 cm	10	18.7	1.9	51
Surface 4 cm	10	D5 = 3	3.3	4.5
Needle	D8 = 8	D13 = 2.2	3.6	1.1

### Flat Applicators

**Figure 6:**
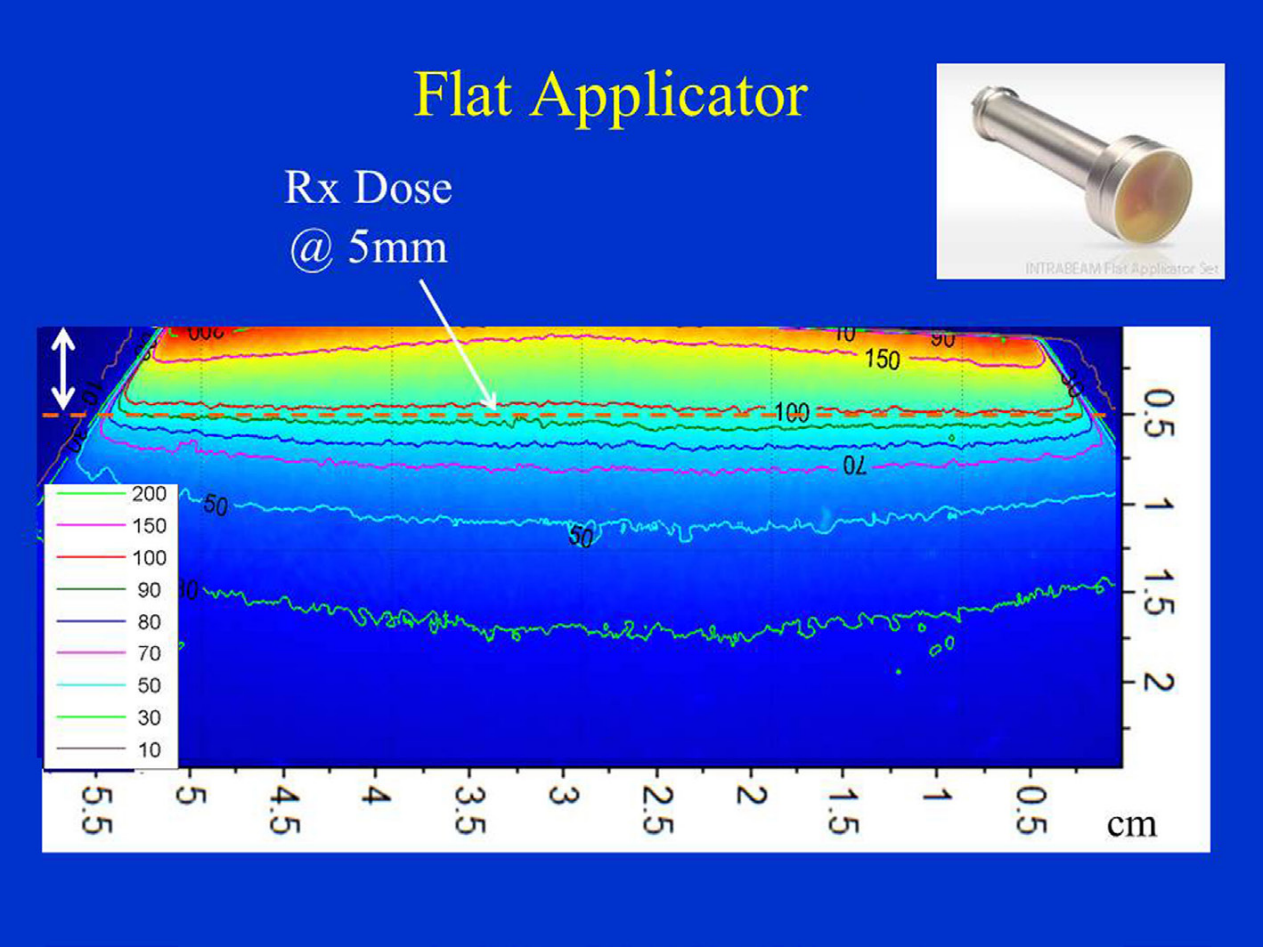
Dose distribution produced by a 4 cm flat applicator. Dose is prescribed at 5 mm depth in phantom. Also indicated is 5 mm superficial layer used to evaluate dose homogeneity. Applicator shown in inset.

**Figure 7:**
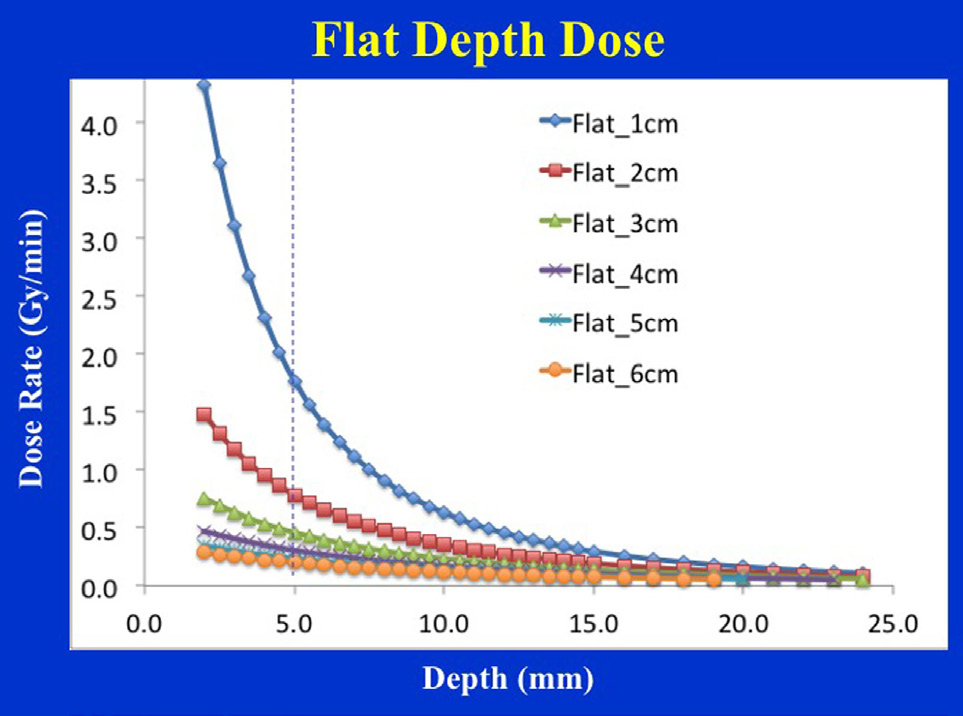
Flat applicator depth-dose (DD) profiles for a range of diameters (1–6 cm) as measured with an ion chamber. Larger applicators are characterized by superior dose homogeneity, lower surface dose, smaller output factor, and longer treatment times. Notice a lack of measured data at shallow depths (<2 mm) due to the chamber design.

**Figure 8:**
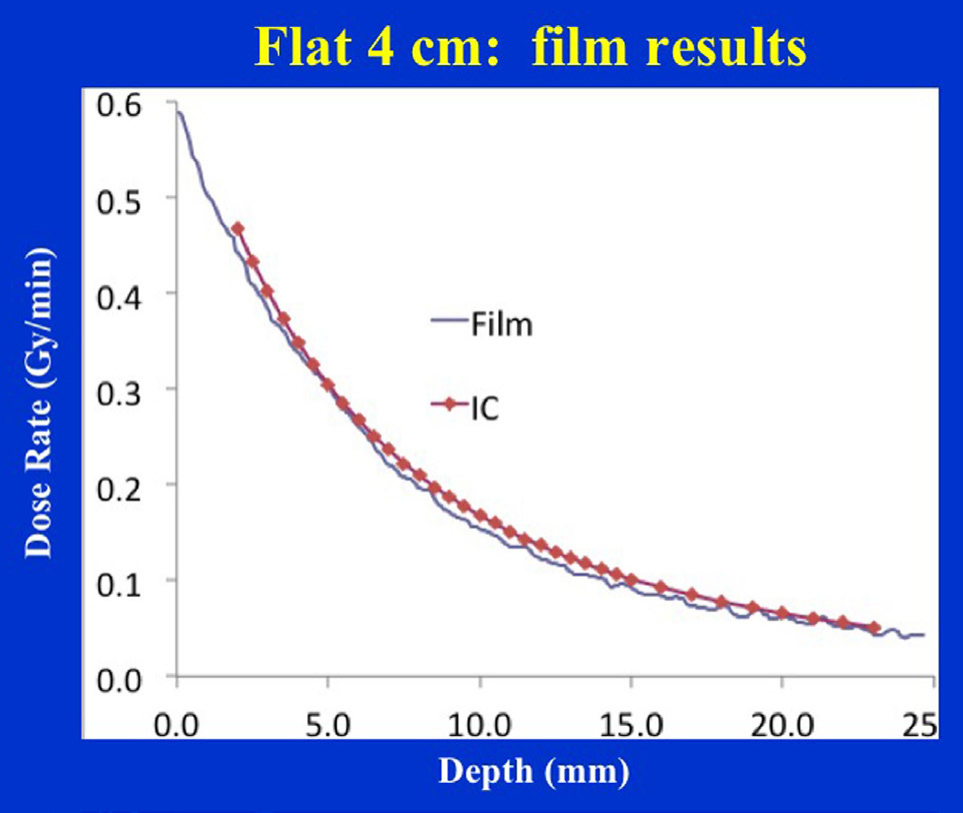
Film vs. ion chamber depth dose comparison for a 4 cm diameter flat applicator.

Figure 6 shows the dose distribution produced by a 4 cm diameter flat applicator. The dose uniformity (or flatness) perpendicular to the beam direction is greatest at the prescription depth of 5 mm with the dose being less uniform at other depths. At shallower depths (<5 mm from the skin-surface), “horns” in dose profiles corresponding to higher dose values are seen at points away from the central axis. For deeper depths (>5 mm), the opposite effect is observed: the measured dose is greatest along the central axis but tapers-off away from it. For a prescription dose of 10 Gy at 5 mm depth, 19.4 Gy would be delivered to the skin-surface corresponding to a dose-homogeneity, DH of 1.94 along the beam direction within 5 mm thick surface layer. Typical treatment time is approximately 30 min. Larger applicators require longer treatment times, but result in a lower surface dose (Ds) and superior dose-homogeneity. Figure 7 shows the dose fall-off with depth (DD) in water for various flat applicators ranging in diameter from 1 to 6 cm. Several interesting features of this figure are worth noting. First, small applicators are associated with (a) large surface (or skin) dose, (b) shorter treatment times, and (c) lower DH. Second, Figure 7 shows a lack of measured data for depths <2 mm. This is caused by the design of the parallel-plate ion-chamber used for dose measurements. The effective point of measurement for this chamber is located at its entrance (inner) wall, which is ~2 mm below the chamber’s outer surface. As shown in Figure 2C, the chamber wall thickness is 0.22 mm and the chamber is protected inside a 1 mm thick waterproof sleeve (Figure 2A) with an air gap of 0.5 mm between the sleeve and the chamber wall. Thus, a separation of 1.72 mm between the inner wall of the chamber and its outer surface represents the region where dose cannot be measured. To obtain missing data points in the superficial region, we repeated DD measurements with EBT3 films oriented parallel to the beam direction. Figure 8 shows excellent agreement between film and ion chamber results for a 4 cm flat applicator.

### Surface Applicators

**Figure 9:**
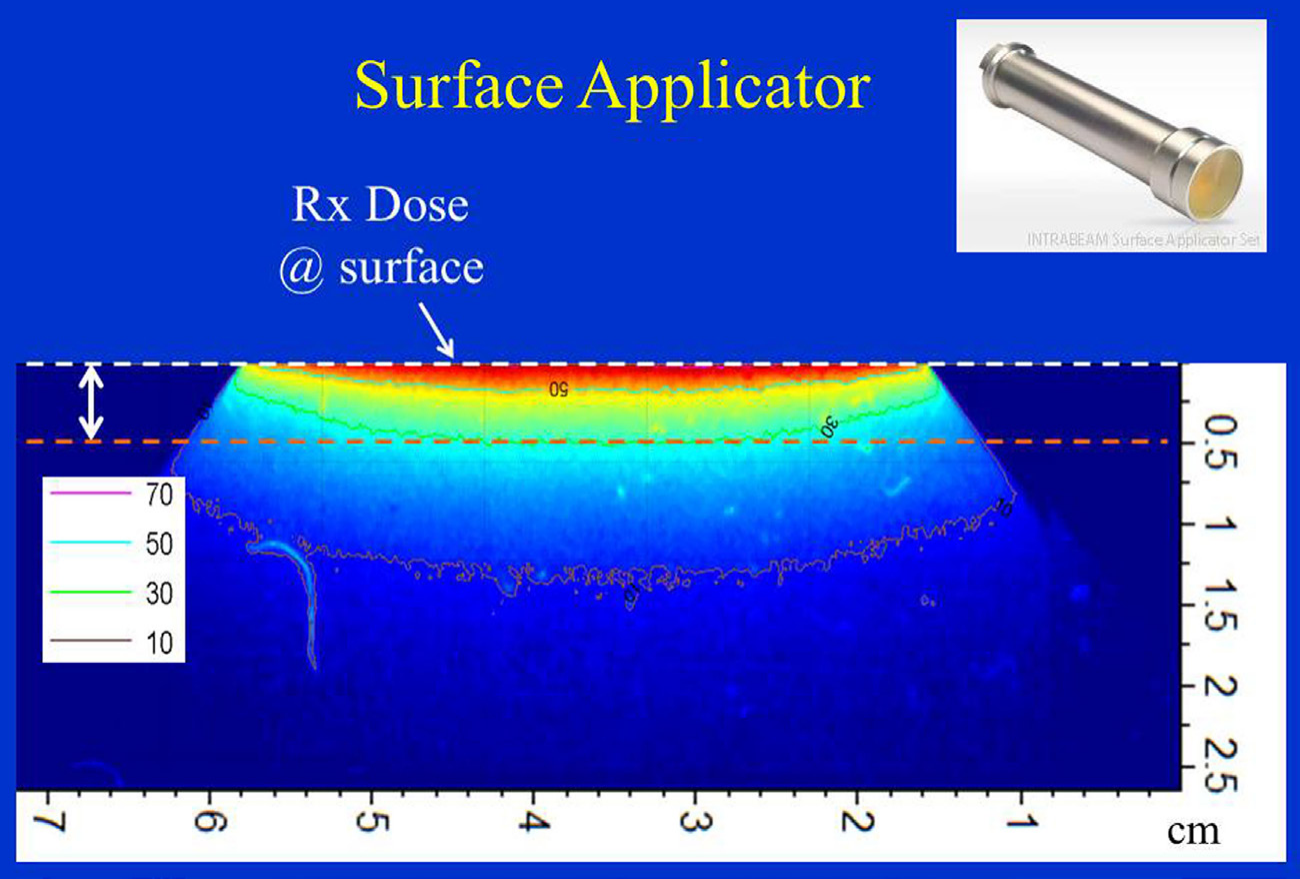
Dose distribution from a 4 cm surface applicator. Dose is prescribed at the surface of the phantom. Also indicated is 5 mm superficial layer used to evaluate dose homogeneity. Applicator shown in inset.

**Figure 10:**
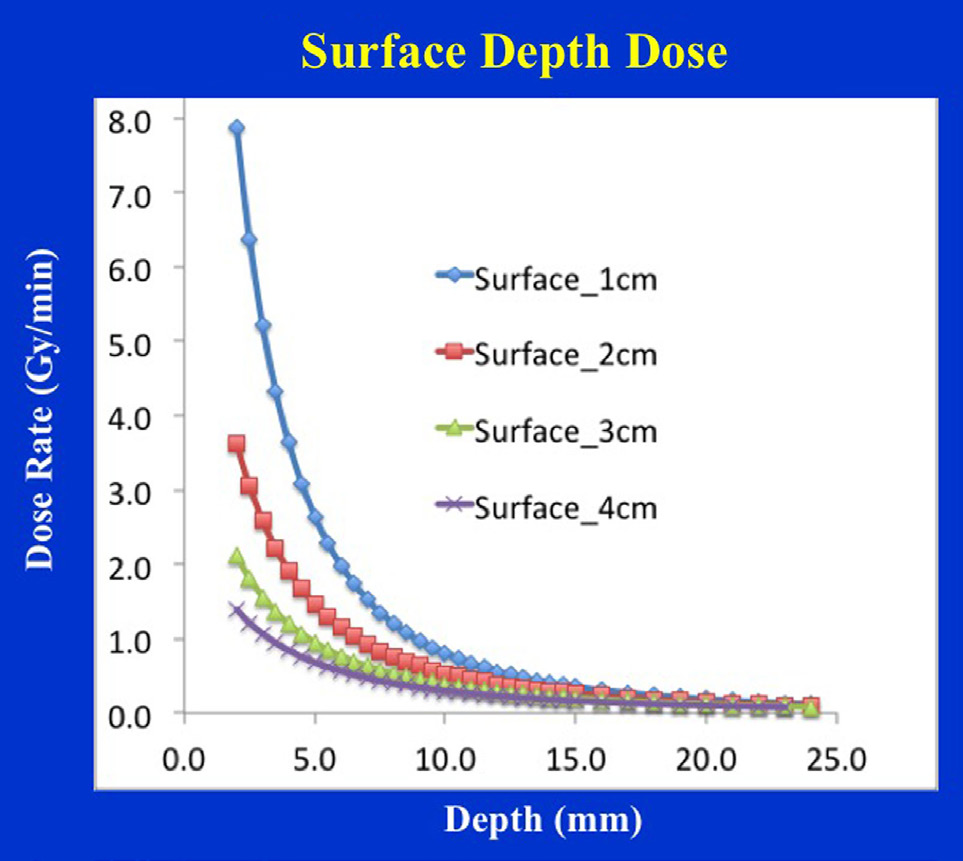
Surface applicator depth-dose (DD) profiles for a range of diameters (1–4 cm). Larger applicators are characterized by superior dose homogeneity, smaller output factor, and longer treatment times. Notice a lack of measured data at shallow depths (<2 mm) due to the chamber design.

**Figure 11:**
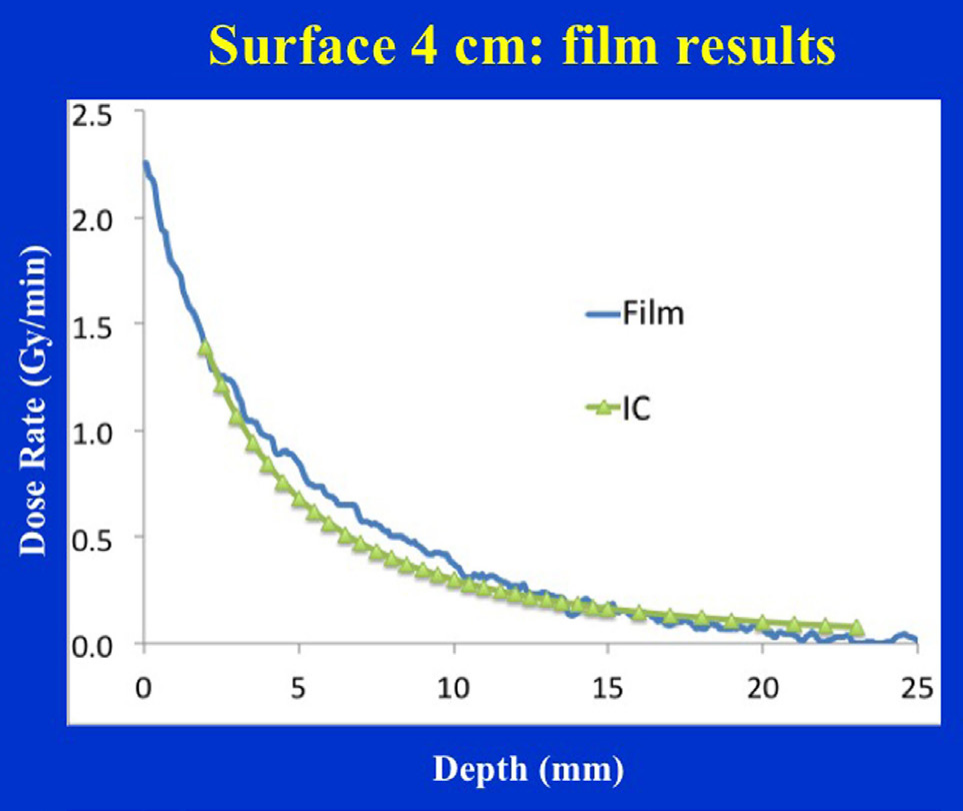
Film vs. ion chamber depth dose comparison for a 4 cm diameter surface applicator. Discrepancy between film and ion chamber data could be related to X-ray spectral changes resulting from steep dose gradient associated with surface applicators.

Figure 9 shows dose distribution from a 4 cm diameter surface applicator. The prescription dose in this case is at the applicator surface and a rapid dose fall-off is observed with depth. The dose is highest along the central-axis and tapers off-axis. For a prescription dose of 10 Gy at the applicator (or skin) surface, a treatment time of 4.5 min is required. This corresponds to a surface dose-rate (Ds) of approximately 2.2 Gy/min. At a depth of 5 mm, the dose reduces to only 3 Gy for a DH = 3.33. Again, larger diameter surface applicators will require longer treatment times but produce superior DH and lower Ds. Figure 10 shows the dose fall-off with depth for various surface applicators. To recover missing dose data points at the shallower depths (<2 mm), doses were re-measured with calibrated EBT3 films and the results are shown for the 4 cm-surface applicator. Good agreement between ion-chamber and film doses is seen (Figure 11). The discrepancy between film and chamber dose is probably due to X-ray spectral changes and rapid dose fall-off with surface applicators.

### Needle Applicators

**Figure 12:**
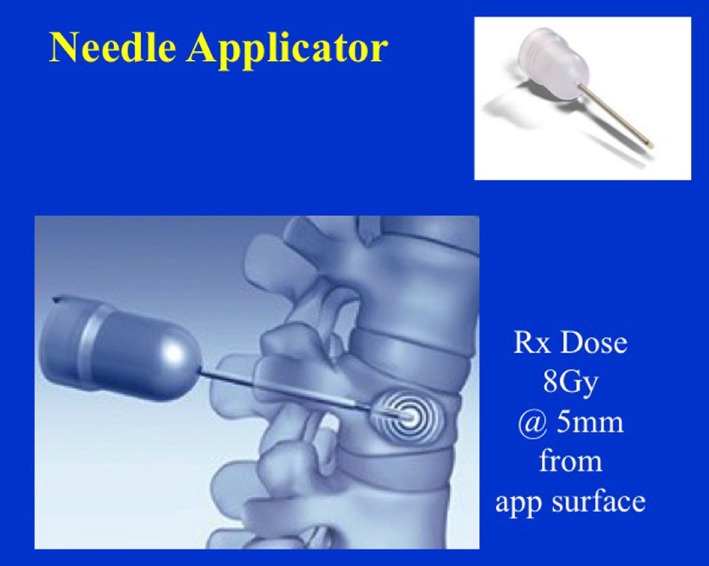
Dose distribution from a needle applicator. Dose is prescribed at 5 mm from the applicator surface or 8 mm from the source. Needle applicator shown in inset.

**Figure 13:**
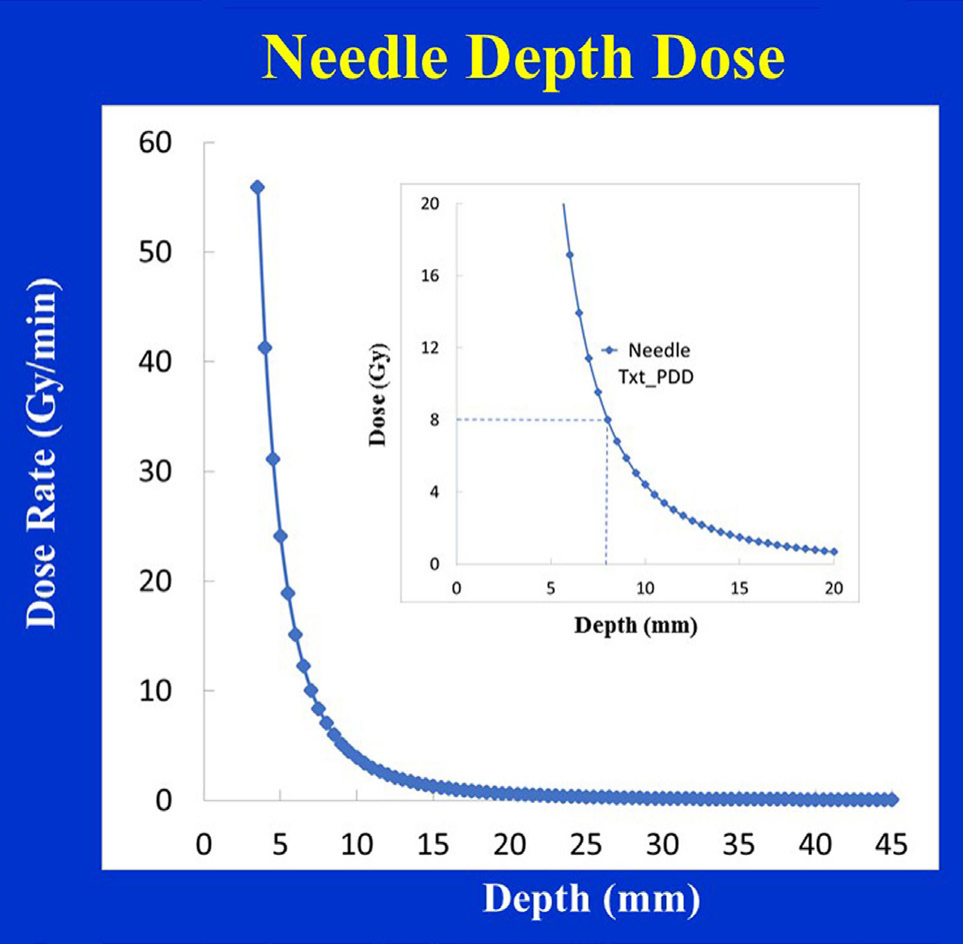
Depth-dose (DD) profiles for the Needle applicator (diameter 4.4 mm). Inset figure shows a prescribed dose of 8 Gy delivered at 8 mm from the source.

Figure 12 shows dose distribution produced with a needle applicator. Typically, 8 Gy prescription dose is given at 5 mm distance from the needle applicator surface to spine metastases for a treatment time of over a minute. Owing to the close-proximity of the prescription dose point to the XRS source, a rapid dose fall-off with depth is observed (Figure 13). A look-up table (available from Zeiss, Inc.) is used to determine treatment time for desired prescription dose.

Table 1 summarizes main dosimetric results for various IORT applicators.

## Discussion

Intraoperative radiotherapy has shown clinical utility in a variety of treatment sites outside breast: intracranial (20), head and neck (21, 22), abdomen (23), pelvis (24, 25), spine (26, 27), and skin (28). Compared to 3-d conformal radiotherapy, the main advantages of IORT lie in its steep dose-fall off and the ability to give a large dose to target volume while limiting dose to nearby organs-at-risk (OARs). Successful IORT requires a multi-disciplinary team of nurses, anesthesiologists, radiation oncologists, surgeons, and medical physicists. Also needed are quality assurance checks for safe and effective IORT delivery and a detailed understanding of each applicator’s dose distribution. Since April 2014, our group has treated 73 IORT patients for a variety of treatment indications: breast (35 patients), H&N (23 patients), abdomen/pelvis (13 patients), and spine metastasis (2 patients).

The choice of IORT applicator depends on the treatment site and the extent of the disease. For example, breast IORT is commonly delivered using a spherical applicator with the prescription dose at the outer surface of the applicator (or inner-cavity surface). Flat and surface applicators provide a uniform planar dose as in the H&N and abdomen/pelvis regions. At our institution, H&N IORT has been delivered with flat applicators; however, both flat and surface applicators have been used in the treatment of abdomen/pelvis targets. The needle applicator has been designed for kypho-IORT of spine metastasis.

Each IORT applicator presents a unique dosimetric challenge and learning curve, due attention to which is essential for safe and effective treatments. Prior to their clinical use, dosimetric parameters: surface dose (Ds), depth dose profiles (DD), treatment times, etc., for each applicator must be measured and validated. These data, as a lookup table, can be helpful in performing quality assurance or independent checks of treatment times used for patient treatments. In addition, room survey measurements must be performed for each applicator to assess doses received by the OR personnel during IORT and to ensure they are within safe-limits.

The maximum dose with a spherical applicator is at the applicator surface. In general, radial dose fall-off with depth is sharper with smaller applicators. The use of small applicators may, therefore, result in greater skin sparing but a less uniform target dose. Small applicators are also associated with shorter treatment times. Using too small an applicator size, however, could cause air-gaps between the applicator and the surrounding cavity, thereby compromising treatments. Studies have shown significant attenuation of low energy photon spectrum in the presence of tissue inhomogeneities (29). These may produce unacceptably large variation in PTV dose. Based on film and ion chamber measurements, our group showed a 16% dose enhancement when a 2 mm layer of tissue is replaced by air and a 58% dose reduction when it is replaced by bone (29). This is further exacerbated by the above noted rapid variation in target dose with depth; for example, a 3.5 times reduction in dose within 1 cm thick shell surrounding a 4 cm diameter breast applicator. Therefore, Bouzid et al. have recommended CT-based treatment planning with Monte Carlo dose calculations for improved prescription and assessment of delivered IORT dose (30). With CT based planning, any concerns related to a lack of target coverage and/or OAR sparing can be addressed by the planner.

**Figure 14:**
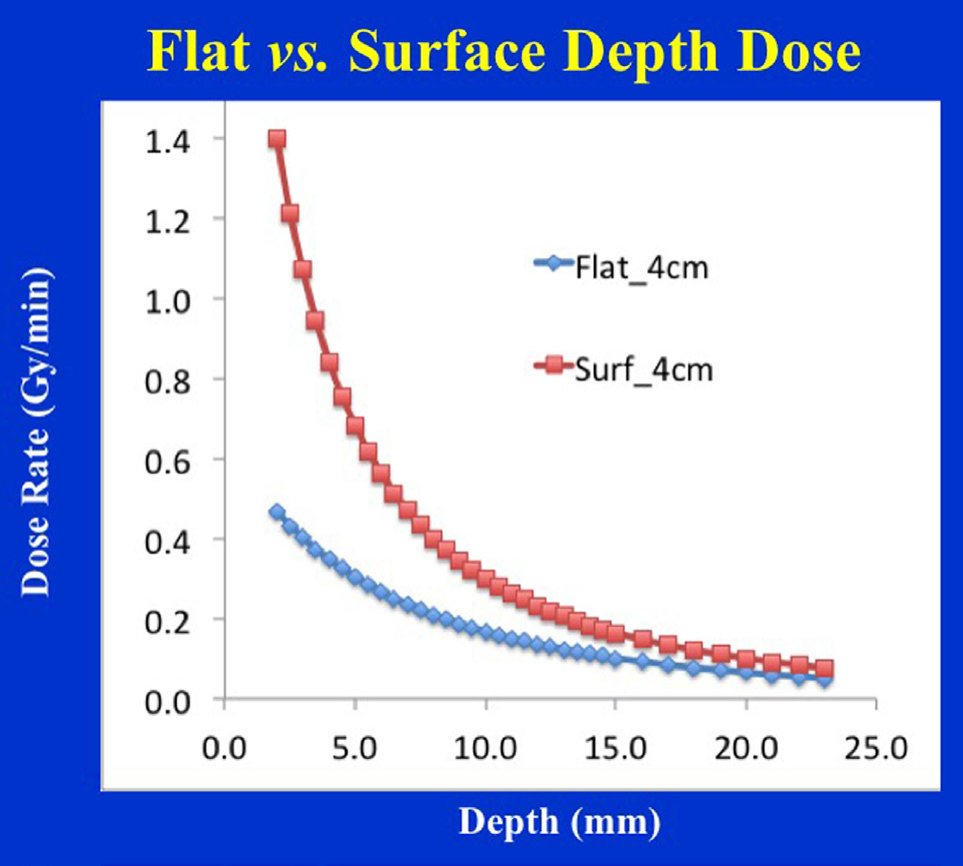
Depth-dose profile comparison for 4 cm diameter flat vs. surface applicators. Notice the steep dose-fall of with depth for the surface applicator resulting in inferior dose homogeneity, larger output factor, and shorter treatment times.

A flat applicator is used when a uniform dose at a given depth is desired in tissue (typically 5 mm depth from skin-surface). With an increase in applicator diameter, the dose-rate decreases, the treatment time increases, but the DH is improved in the shallow regions. These changes are most dramatic for smaller applicators (<3 cm diameter). In general, larger applicators produce the most homogeneous dose. Similar depth-dose variation is observed for surface applicators but the effect is more pronounced (Figure 14). A concern with the use of flat/surface applicators is the skin/surface dose, which could be a limiting factor in some treatments. It is important to note that very small applicators (≤2 cm diameter) may yield relatively high surface dose; therefore, one must select the largest possible applicator size that is compatible with the treatment area.

Due to large prescription doses used with IORT, skin-toxicity can be a big concern. We have investigated the use of SURGICEL^®^ (Ethicon, Inc., Johnson and Johnson Health Care, Somerville, NJ, USA) to reduce surface dose. A 1 or 2-mm layer of SURGICEL^®^ may be used to reduce skin toxicity (lower skin dose by up to 30%).

In summary, the dosimetric results presented here pertain to INTRABEAM IORT and associated applicators only. Furthermore, the results reported in this study are for guidance purposes only and must be validated by each institution with their own equipment prior to the clinical implementation of IORT.

## Conclusion

Intraoperative radiotherapy may be delivered with a variety of treatment applicators. Selection of appropriate applicator is important for safe, efficient, and effective delivery of IORT. The dosimetric results from this study should help design IORT treatments to assure optimized target coverage and reduced normal tissue exposure. These results may also be used in designing an effective IORT QA program including secondary check of treatment times.

## Author Contributions

AS participated in study design, data measurement, and manuscript preparation. BE, WS, and TT contributed clinical data and recommended applications and reviewed manuscript.

## Conflict of Interest Statement

The authors declare that the research was conducted in the absence of any commercial or financial relationships that could be construed as a potential conflict of interest.
